# Supplementary material for: The Role of Annexin A1 in DNA Damage Response in Placental Cells: Impact on Gestational Diabetes Mellitus
Source: Int J Mol Sci. 2023 Jun 15;24(12):10155. doi: 10.3390/ijms241210155 (PMC10299641; doi:10.3390/ijms241210155)
Supplement: Supplementary file 1 [file ijms-24-10155-s001.zip › ijms-2418446-supplementary.pdf]

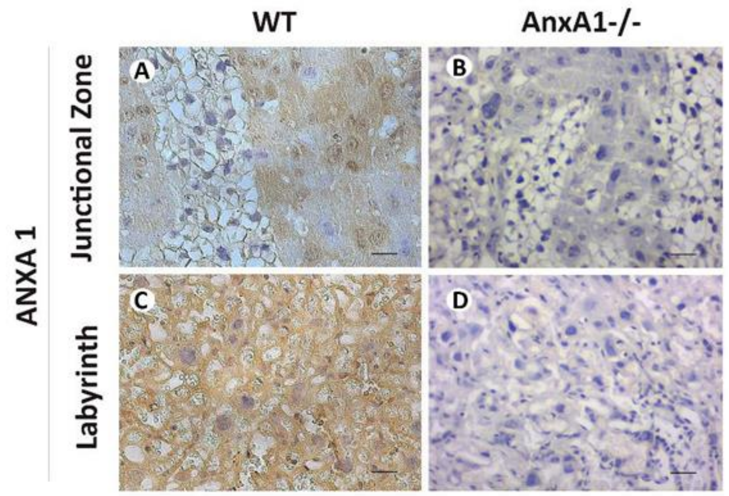

Figure S1. Immunolocalization of ANXA1 in placenta sections from WT (A and C) and AnxA1−/− (B and D) animals. (A-C) Placental cells reactive to ANXA1 are found in both junctional and labyrinthine placental zones with a similar pattern of immunoreactivity. (B and D) No AnxA1 reactive placental cells were observed in AnxA1−/− group, confirming the ANXA1 knockout model. (A-D) Immunoperoxidase and hematoxylin counterstaining; Bars = 50 µm; n=3/group.

Table S1: Histological analysis of ND and GDM human placentas

	ND (n=10)	GDM (n=10)
Fibrin deposition score	1	4
Syncytiotrophoblast knots score	0	3

Semiquantitative scoring system for histological analysis of ND and GDM human placentas: Score 0: normal; 1: event occurring in less than 25% of the field; 2: 25 to 50% of the field; 3: 51% to 75% of the field and 4 more than 75% of the field. Adapted from Meyerholz & Beck (2018) [39].
